# Endomicroscopic analysis of time-dependent and pressure- dependent recruitment of subpleural alveoli

**DOI:** 10.1186/cc13462

**Published:** 2014-03-17

**Authors:** H Runck, D Schwenninger, S Schumann, J Haberstroh, J Guttmann

**Affiliations:** 1 University Medical Center, Freiburg, Germany

## Introduction

We used transthoracic endoscopy [1] to continuously record images of subpleural alveoli during recruitment manoeuvres and different steady plateau pressures between the manoeuvres.

## Methods

We investigated two groups of 13 rats each. Animals were ventilated with ZEEP or PEEP of 5 mbar, FiO_2 _of 1.0 and tidal volume of 10 ml/kg. A double low-flow manoeuvre was designed, consisting of two consecutive low-flow manoeuvres with a peak pressure of 30 mbar, interrupted by a 5-second plateau phase at different pressures (2, 4, 8 and 12 mbar). Alveolar size at the peak pressures and during the plateau levels was analyzed from the recorded videos frame by frame. Therefore the alveolar outline of 10 alveoli was marked manually and the outlined area was calculated [2]. Compliance of tidal breaths before and after the manoeuvres was calculated using two-point compliance.

## Results

In both groups, analysis of the alveolar area revealed that alveolar size at the second peak of the manoeuvre did not differ significantly compared with the first peak (100.97% in ZEEP group, 102.37% in the PEEP group). During the plateau phases there was a slight increase in alveolar size at higher plateau pressures (slope of linear regression at plateau 4 mbar: 0.1 %/500 ms for ZEEP group, 0.18./50 ms for PEEP group; at plateau 8 mbar: 0.42%/500 ms for ZEEP group, 0.565%/500 ms for PEEP group). After the manoeuvres, compliance increased to 137.73% in the ZEEP group and 119.91% in the PEEP group.

## Conclusion

In the healthy lung, once recruited, alveoli stay stable in size over wide pressure ranges. Further recruitment manoeuvres do not lead to further increase of alveolar size, but increase of compliance. During plateau phases, alveolar size increases dependent on pressure. This leads to the assumption that recruitment is not only pressure dependent but also time dependent.
